# Prebiotic effects of *Talinum triangulare* and *Mangifera indica* on slow growing broiler chickens (SASSO)

**DOI:** 10.1016/j.heliyon.2024.e25557

**Published:** 2024-02-01

**Authors:** Bruno Dossou Sodjinou, Pierre Faya Leno, Germaine Millimono, Sêmihinva Akpavi, Kokou Tona, Frédéric Makpondji Houndonougbo

**Affiliations:** a Regional Center of Excellence on Poultry Sciences (CERSA), University of Lome, Lome, Togo; b Laboratory of Botany and Plant Ecology (LBPE), University of Lomé, 01 BP 1515, Lomé 01, Togo; c Faculty of Agronomic Sciences (FSA), University of Abomey-Calavi, Cotonou, Benin

**Keywords:** Prebiotic, Talinum triangulare, Mangifera indica, Broiler

## Abstract

**1:**

The study aim was to evaluate the prebiotic effects of *Talinum triangulare* and *Mangifera indica* used on slow growing broiler chickens.

**2:**

Three hundred and sixty (360) slow-growing chicks of four weeks of age and similar weight were selected and divided into four (04) treatments (Positive Control, Negative Control, 2 % *T. triangulare* and 2 % *M. indica*) of 6 replicates with, fifteen (15) chicks per replicate, which made ninety (90) chicks per treatment.

**3:**

At 12 week age, blood sample and cecal content were taken from 6 chickens per treatment to determine heamatological profile and fermentation parameters (Short Chain Fatty Acid). The data obtained were submitted to one-way analysis of variance (ANOVA) using the software R version 3.6.2 (R Core Team, 2019).

**4:**

Results showed that growth performance, haematological parameters, acetic, butyric, valeric and caproic acids were similar between broilers fed with the leave powders and the positive control treatment. However, broilers fed with *Talinum triangulare* and *Mangifera indica* powders showed a lower mortality rate, compared to the negative and positive control treatments. Moreover, broilers fed with the leave powders showed significantly higher (p < 0.05) formic acid concentration than the other treatments.

**5:**

*Talinum triangulare* and *Mangifera indica* leaves could have prebiotic properties because they stimulated the production of short-chain fatty acids that keep animals healthy.

## 1 Introduction

Genetic development, improved feed use and good health outcomes involve antibiotic use as therapeutic agents in poultry industry [1,2]. Antibiotics have contributed significantly to intensive poultry production, but an increase in antibiotic-resistant strains of bacteria has been observed in poultry farms among other livestock species [3]. The high incidence and growing frequency of antibiotic resistance of these bacteria in poultry farms constitute a public health hazard [4,5]. These antibiotic-resistant bacteria strains enter the food chain and pose health risks to the consumer [6]. Additionally, the increased cost of production due to the scarcity of food in developing countries is a constraint to the development of poultry farming [7]. Protein and energy components account for more than 90 % the total nutrient requirements in poultry rations [8]. These conventional protein and energy components are expensive, because of competition between humans and animals. In response to these global problems, much research has been carried out to attain a satisfactory level of production.

It has been found that the use of probiotics [9], prebiotics [10], symbiotics [11] and phytogens [11,12] in poultry farming can alleviate the problems of antibiotics and the high cost of poultry inputs. Prebiotics were the focus of some study because of their mechanisms of action on poultry gut flora [13] and their physiological benefits on poultry health [14]. Prebiotics are derived from a wide variety of herbs, trees, spices and derived products [11,15,16]. Supplementation with the plant-derived isoquinoline alkaloid improved growth performance, protein catabolism and intestinal barrier functions [17]. In addition, essential oils improve poultry production by increasing the activity of digestive enzymes, lowering the number of fermentation products, lowering the number of pathogens, improving nutrient digestion, enhancing intestinal accessibility of important nutrients, and enhancing antioxidant capacity and immune function [18,19].

*Mangifera indica* and *Talinum triangulare* are medicinal plants belonging to the Anacardiaceae and Portulaceae families respectively [20,21]. Studies in the sub-region of west Africa on these plants show that all parts of these plants are used mainly in phyto-medicine [22,23]. In the poultry sector, several studies have been carried out on the incorporation of dry leaves of *T. triangulare* and *M. indica* and their extracts at different rates and/or doses [[24], [25], [26]]. All these studies were carried out on fast-growing broilers and laying hens with satisfactory results. In fact, Sasso chickens are a slow-growing genetic type renowned for their thermotolerance in tropical areas are similar to indigenous chickens, most of which are slow-growing [27]. In addition, the quality of the meat and the good flavour [28] lead to more interest of consumers in these chickens [29].

Recent research [30] reported that *Mangifera indica* and *Talinum triangulare* exist in Togo and are used in poultry farming for nutritional needs and to treat certain poultry diseases. Despite the importance of these two plants to poultry, scientific data on them are unavailable or non-existent in Togo. This study aimed at evaluating the prebiotic effects of *Mangifera indica* and *Talinum triangulare* use in poultry farming in Togo.

## 2 Materials and methods

### 2.1 Ethics statement

This study was carried out in strict accordance with the recommendations of the Guide for the Care and Use of Experimental Animals of the University of Lome, Togo. The protocol was approved by the Ethics of Animal Experimentation Committee of the same University. All efforts were made to minimize discomfort to the birds.

### 2.2 Experimental design

A total of three hundred and sixty (360) chicks of the SASSO strain were reared for 12 weeks of age. During the first three weeks of age, all chicks were fed starter feed. At the fourth week of age, after weighing, three hundred and sixty (360) unsexed broiler chicks of similar weight were selected and randomly allocated to four (04) treatments of 6 replicates of 15 birds each. The feeding trial started at 5th week of age and lasted eight (8) weeks. The broilers were distributed according to the following experimental treatments:

Negative control (T-): control feed + prophylaxis program without antibiotics.

Positive control (T+): control feed + prophylaxis program with antibiotics.

2 % *Talinum triangulare*: feed + 2 % *T. triangulare* + prophylaxis program without antibiotic.

2 % *Mangifera indica*: feed +2 % *M. indica* + prophylaxis program without antibiotic.

### 2.3 Collection and preparation of *Mangifera indica* and *Talinum triangulare* leaves

Leaves of *Mangifera indica* and *Talinum triangulare* were collected respectively at the University of Lomé (6°10′24.41″N and 1°12′58.63″E) and Badja (6°22′15.65″N and 0°58′07.35″L). These species were identified by the research team of the Laboratoire de Botanique et Ecologie Végétale (LBEV) of the University of Lomé. The specimens of each plant were deposited at the Herbarium of the University of Lomé under the numbers TOGO15762, TOGO 15778 respectively. They were dried within 5–14 days respectively in the shade in an air-conditioned room at 19 °C at the CERSA laboratory. After drying, *Mangifera Indica* and *Talinum triangulare* leaves were ground into powder (see Table 1 for the phytochemical and nutritional composition of these plants).

**Table 1:** Phytochemical and nutritional composition of leaves of *Mangifera indica* and of *Talinum triangulare*.

Phytochemical composition leaves of plants	*T. triangulare*	*M. indica*
Alkaloïd (%)	0.47	0.3
Flavonoïd (%)	0.26	1.05
Tannins (%)	0.36	0.97
Sterol (%)	0.03	–
Terpenoïd (%)	0.42	–
Total phenol (%)	0.02	1.34
Saponins (%)	0.68	1.24
Cardiac glycosides (%)	–	0.13
**Nutritional composition leaves of plants**	***T. triangulare***	***M. indica***
Fat content (%)	0.34	4.31
Ash content (%)	8.03	11.49
Carbohydrate (%)	53.99	30.6
Crude protein (%)	19.97	18.59
Crude fiber (%)	8.97	13.99
Magnesium (%)	0.021	0.098
Potassium (%)	0.061	0.589
Manganes (%)	0.0001	0.003
Cuivre (%)	0.0006	n
Fer (%)	0.002	0.368
Zinc (%)	0.009	0.014
Phosphore (%)	0.0037	0.48

Sources [22]: [37]; [32]; n: non detected.

## 3 Experimental feeds

Chicks received diets containing (3100 kcal/kg of ME, 21 %BP) for three weeks of age. During the experimental phase, the broilers were fed a feed appropriate for each breeding phase (see Table 2 for feed composition). Water and feed were served *ad libitum*. From the fourth week onwards the change in diet was made gradually over four days in the proportions of ¼, ½, ¾ and 1 respectively.

**Table 2:** Feed and nutrient compositions of diets used per treatment, at different phases of production.

Feeds ingredients	Feed composition						
	Starter phase	Growing phase			Finisher phase		
	Control	Control	2 % Tal	2 %Man	Control	2 % Tal	2 %Man
Maize (Kg)	58.6	54	54	54	58	58	58
Fish flour 40 % (Kg)	8.5	10	10	10	11	11	11
Soybean cake (kg)	26	22	22	22	21	21	21
Wheat offals (Kg)	3	11	9	9	8	6	6
*T. triangulare* (Kg)	0	0	2	0	0	2	0
*M. indica*(Kg)	0	0	0	2	0	0	2
Oyster shell(Kg)	0.8	0.9	0.9	0.9	0.9	0.9	0.9
Premix broiler (Kg)	3	2	2	2	1	1	1
Methionine (Kg)	0.1	0.1	0.1	0.1	0.1	0.1	0.1
Lysine (Kg)	0.01	0.01	0.01	0.01	0.01	0.01	0.01
Nutrients							
Enery (Kcal/kg)	3110.71	2975.63	2943.25	2943.33	3028.65	2996.28	2996.28
Crude protein (Kg)	21.58	20.83	20.99	20.77	20.26	20.41	20.19
Lysine (Kg)	1.24	1.16	1.15	1.15	1.15	1.14	1.13
Methionine (Kg)	0.49	0.55	0.55	0.55	0.53	0.53	0.53
Calcium (Kg)	0.91	0.96	0.98	1	0.97	0.99	1.01
Phosphorus (Kg)	0.66	0.72	0.7	0.75	0.68	0.66	0.71

Supplied per kilogram of diet; Rétinol: 15 000 IU; Chrolécalciférol: 5000 IU; α-tocophérol: 100 mg; phytoménadione: 5 mg; Cobalamine: 0.02 mg ; , Niacin: 100 mg ; , Folic acid: 3 mg; Biotin: 0.3 mg; 2%Tali = 2 % T. triangulare; 2%Man = M. indica.

## 4 Data collection

During the experimental period, feed was quantified per day, and left over feed was weighed on a weekly basis. Mortality was recorded on daily basis and body weight of birds were recorded at the end of each week. Feed consumption (FC), weight gain (WG) and feed conversion ratio (FCR) were calculated as follows:

$$\text{Feed}\;\text{consumption}\;\left(\text{FC}\right)=\frac{amount\;of\;feed\;given-remaining\;feed\;}{7\times n}\left(g/broilers\right)$$

With: n = number of broilers

$$\text{DWG}=\frac{\text{weight}\;\text{at}\;\text{the}\;\text{ended}\;\text{week}\;-Weight\;at\;the\;started\;week\;}{7\times n}\;\left(g/j\right)$$

With: DWG = Daily Weight Gain

$$\text{Conversion}\;\text{ratio}\;\left(g\;\text{of}\;\text{feed}/g\;\text{of}\;\text{meat}\;\right)\;\frac{FC\;}{\text{DWG}}$$

At the end of the rearing period, 1 chicken per replicate (6 chickens per treatment) was selected according to the average weight of the chickens in each cage. The blood samples were collected into tube containing ETDA (Ethylene Diamine Tetra-Acetic acid) as anticoagulant from the wing vein of each chicken, using a sterilized disposable syringe and needle, to determine haematological parameters. The birds were slaughtered by severing the jugular vein and the cecal contents of each chicken were collected in an Eppendorf tube. The latter was used to determine the fermentation parameters (Short Chain Fatty Acid) of the chicken cecum.

### 4.1 Haematological parameters

Haematological indices were analyzed by the following methods: Red blood cell (RBC) and white blood cell (WBC) counts were determined by the Neubauer haemocytometer method. The haemoglobin (Hb) concentration was determined according to the method described by using cyanomethaemoglobin. The mean corpuscular volume (MCV), mean corpuscular haemoglobin (MCH) and mean corpuscular haemoglobin concentration (MCHC) were determined on the basis of the formulae of [31].

Dosage of the Short Chain Fatty Acid (SCFA) parameters.

### 4.2 Sample treatment

Cecal SCFA concentrations were measured by gas chromatography coupled with mass spectrometry. Briefly, cecal contents (50 mg) were homogenized in 200 μL of 24 % metaphosphoric acid using a vortex mixer for 15 min and centrifuged at 5000×*g* for 5 min at 4 °C. The supernatants were transferred to headspace vials (10 mL) and made up to 5 ml using deionized water. The vials were then crimped and transferred to the headspace sample for analysis.

### 4.3 7697A headspace parameters

The samples were introduced into the GC by means of a headspace sample. Vials pressurization gas used was nitrogen gas and helium were used as the carrier gas.

Oven temperature was set at 80 °C; Loop temperature was set at 85 °C; Transfer line temperature was set at 120 °C; Vial equilibration duration was set at 10 min; Fill pressure was set at 15psi.

#### 4.3.1 7890A GC parameters

Sample were analyzed using Agilent 8860 GAS chromatograph equipped with a flame ionization detector (FID), fitted with a DB-624 capillary column coated with 6 % cyanopropyl/phrnyl, 94 % polydimethylsiloxane (30 m length × 0.53 mm diameter × 3 μm film thickness) (Agilent technologies).

The sample was injected in split mode (1:1) at an injection temperature of 250 °C, at a pressure of 4.227 psi and a total flow of 0.6 ml/min. The oven was initially programmed at 35 °C (1min) and then ramped at 10 °C/min to 200 °C. FID temperature was 300 °C with hydrogen: Air flow at 30 ml/min: 300 ml/min, helium was used as make-up gas at a flow of 18 ml/min.

#### 4.3.2 Calibration procedure

VOCs standard, 2000 ppm containing 10 SCFA components was purchased from AccuStandard (USA). Three (03) point serial dilution calibration standards (0.05, 0.2, 0.5 mg/l) were prepared from the stock and to calibrate the GC.

### 4.4 Statistical analysis

Data were analyzed with R software, version 3.6.2 (12/12/2019). One-way ANOVA was the test performed on data such as: feed consumption, feed conversion ratio, data of cecal fermentation parameters and haematological parameters. For mortality rates, the beta family GLM procedure performed. The curves were made using Graph pad prism software, version 8.0.0.288 (28/08/2021). Significant differences (p < 0.05) among treatment means were determined using Student Newman Keuls (SNK) multiple range test.

## 5 Results

Feed Intake (FI), Body weight (BW), Feed Conversion Ratio (FCR) and mortality rate (MR) of slow growing broilers.

Table 3 shows the prebiotic effect of *T*. *triangulare* and *M. indica* on feed intake, body weight, feed conversion ratio and mortality rate of Sasso broilers. Feed consumption was similar (p > 0.05) between treatments during the breeding period. Body weight was significantly higher (p < 0.05) in treatments control+, 2 % *M. Indica* and 2 % *T. triangulare*. However, broilers in the control+, 2 % *M. Indica* and 2 % *T. triangulare* treatments had a highly significantly lower feed conversion ratio (p < 0.05) compared to those in the control-treatment. In addition, broilers fed with plant leaves had a highly significantly lower mortality rate (p < 0.05) compared to the other treatments.

**Table 3:** Indicate the prebiotic effect of *T. triangulare* and *M. indica* on feed intake (FI), Body weight (BW), Feed Conversion Ratio (FCR) and mortality rate (MR) of broilers.

Parameters	Treatements				p-value
	Control -	Control +	2 %*T. Triangulare*	2 %*M. Indica*	
FI (g)	73.97 ± 0.47	73.84 ± 0.72	73.98 ± 0.04	73.98 ± 0.04	0.09
BW (g)	1509.19 ± 51.51^b^	2064.56 ± 65.28^a^	1946.76 ± 70.06^a^	1963.28 ± 54.40^a^	***
FCR	4.90 ± 0.51^a^	3.46 ± 0.40^b^	3.56 ± 0.12^b^	3.58 ± 0.20^b^	***
MR (%)	12.63 ± 0.50^a^	3.50 ± 0.66^b^	2.30 ± 0.48^c^	2.47 ± 0.75^c^	***

FI: Feed Intake; BW: Body Weight; FCR: Feed Conversion Ratio; MR: Mortality Rate; Means on the same line with different letters are significantly different (P < 0.05).

### 5.1 Haematological parameters of slow-growing broilers

Table 4 presents the results of the effect of the incorporation of *T. triangulare* and *M. indica* leaf powders on haematological parameters. In general, the results of the haematological parameters of the negative control group were lower than the normal value of each haematological parameter of the birds. However, the values for the positive control, 2 % *T triangulare* and 2 % *M. indica* treatments were within the normal range of haematological values. Statistically, there was a significant difference (p < 0.05) between the red and white blood cell values of the negative control treatment and the other treatments (control+; 2 % *T. Triangulare* and 2 % *M. Indica*).

**Table 4:** Effect of the incorporation of *T. triangulare* and *M. indica* leaf powders on haematological parameters.

Parameters	Traitements				P. value
	Control -	Control +	2 %*T.Triangulare*	2 %*M.Indica*	
WBC (10^−3^/ml)	3.21 ± 0.43^b^	4.13 ± 0.37^a^	4.10 ± 0.13^a^	4.07 ± 0.17^a^	***
RBC (10^3^/ml)	3.51 ± 0.55^b^	4.29 ± 0.60^a^	4.33 ± 0.57^a^	4.24 ± 0.43^a^	*
HB (g/ml)	10.75 ± 1.33	11.86 ± 1.01	11.63 ± 1.20	12 ± 0.70	0.22
MCV (fL)	78.66 ± 10.48	83.83 ± 9.70	85.83 ± 6.41	84.75 ± 5.33	0.46
MCH (Pg)	25.80 ± 0.68	28.93 ± 5.16	28.03 ± 3.07	27.06 ± 3.86	0.47
MCHC (%)	29.26 ± 1.35	32.13 ± 3.76	32.23 ± 3.65	32 ± 5.35	0.48

Means on the same line with different letters are significantly different (P < 0.05).WBC: White Blood Cell; RBC: Red Blood Cell; HB: Hemoblobin; MCV: Mean Corpuscular Volume; MCH: Mean Corpuscular Hemoglobin, MCHC: Mean Corpuscular hemoglobin Concentration.

Prebiotic effect of *T. triangulare* and *M. indica* on the concentrations of short chain fatty acids produced by broilers.

The concentrations of each short-chain fatty acid are presented in Table 5. In general, the production of total short-chain fatty acid was similar (p > 0.05) between the groups that were treated with *T. triangulare* and *M. indica* leaf powders. But compared to the other two treatments (Control - and control+), it was significantly different (p < 0.05). Moreover, the concentration of butyric, valeric and heptanoic acids were highly significant in the positive control, 2 % *T. triangulare* and 2 % *M. indica* treatments.

**Table 5:** Prebiotic effect of *T. triangulare* and *M. indica* on the concentrations of short-chain fatty acids produced by broilers.

Parameters	Treatments				P-value
	Control -	Control +	2 %*T. Triangulare*	2 % *M. Indica*	
Formic acid	4.57 ± 0.08^c^	10.57 ± 0.25^b^	16.72 ± 0.53^a^	17.44 ± 0.47^a^	***
Acetic acid	0^c^	0.04 ± 0.001^b^	0.048 ± 0.002^b^	0.053 ± 0.003^a^	***
Butyric acide	0.035 ± 0.002^b^	0.076 ± 0.002^a^	0.072 ± 0.001^a^	0.075 ± 0.004^a^	***
Valeric acid	0.022 ± 0.002^b^	0.048 ± 0.004^a^	0.055 ± 0.001^a^	0.05 ± 0.001^a^	***
Caproïc acid	0.065 ± 0.003^b^	0.067 ± 0.001^b^	0.078 ± 0.001^a^	0.081 ± 0.002^a^	***
Total SCFAs	4.75 ± 0.09^c^	10.88 ± 0.25^b^	17.05 ± 0.53^a^	17.79 ± 0.50^a^	***

Means on the same line with different letters are significantly different (P < 0.05), SCFAs: Short-chain fatty acids.

## 6 Discussion

The objective of this research was to investigate the prebiotic properties of *T. triangulare* and *M. indica* leaves on Sasso broilers. The results of the growth parameters showed that the feed intake, body weight and feed conversion ratio of the positive control treatment and the treatments that received the plant powder were similar (p > 0.05). But, these results were significantly different (p < 0.05) from the results of the negative control treatment. The results of the growth parameters obtained in the chickens that received the plant powders could be explained by the fact that the *T. triangulare* and *M. indica* plant powders are rich in nutrients such as protein, carbohydrate and minerals [32,33]. *T. triangulare* and *M. indica* dry leaves are good sources of protein as they provide more than 12 % of their calorific value from protein [34]. Vegetable proteins are necessary for blood cell formation, maintenance and growth of connective tissues, skin and muscles [35,36]. In addition, phytochemical studies on these plants show the presence of several secondary metabolisms [23,37]. These biomolecules present in these plants are phenols, alkaloids, flavonoids, tannins, coumarins, terpenoids, saponoids, sterols … etc [21,37]. Some of them such as tannins and flavonoids have antibacterial properties [23]. However, our findings were similar to those obtained by Refs. [24,26] who worked on *T. triangulare* and *Mangifera indica* respectively. They concluded that the growth parameters of broilers are similar between the control treatments and those that received the dry leaves of *T. triangulare* and *M. indica*.

Regarding the mortality rate, the results of the treatments that received the leaves of the plants were similar, but they were significantly different (p < 0.05) from the negative and positive control treatments. This can be explained by the fact that *T. triangulare* and *M. indica* have high antioxidant capacity [38]. Indeed, the overproduction of oxygen free radicals leads to oxidative stress, a process that creates several damages to cellular structures and compromises life [39]. Studies have shown that oxygen free radicals are involved in several cardiovascular diseases [40] and acute and chronic liver diseases [41]. Thus, these plants through their antioxidant activities would protect the cells of these broilers from damage caused by oxidative stress, which could explain the very low mortality rates obtained in the treatments that were fed with *T. triangulare* and *M. indica* plants.

The haematological values of the white and red blood cell of the positive control treatment and the treatments that were fed with *T. triangulare* and *M. indica* leaves are similar (p > 0.05). But, the results were significantly different (p < 0.05) between the negative control treatments and the other treatments. According to Ref. [42], all haematological parameter values (WBC, RBC, HB, MCV, HCM, HCM) of the negative control treatment were below normal haematological values, which proves that the broilers in the negative control treatment would not be healthy. Indeed, red blood cells carry oxygen from the lungs to the respiratory tissues and carbon dioxide from the respiratory tissues to the lungs, this is achieved through the presence of haemoglobin [43]. The low values of red blood cells (RBC), haemoglobin (HB) and mean corpuscular volume (MCV) observed in broilers from the negative control treatment demonstrate that the roles of these three constituents (RBC, HB and MCV) of the blood were not fulfilled in these birds. This could expose these birds to anaemia and inflammatory diseases [44]. Furthermore, minerals such as calcium, magnesium and iron are the building blocks of the blood and body of birds [45]. Dry leaves of *T. triangulare* and *M. indica* are rich in minerals [33,37], which could explain the normal results obtained on RBC, HB and MCV in the treatments that received the plant leaves. Our results are different to the findings of [46] on erythrocyte parameters (Hb, RBC, MCV) of rats in the laboratory. These authors showed that the results of erythrocyte parameters in Wistar rats that received ethanolic extracts of *T. triangulare* and *M. indica* leaves were significantly different from the positive control lot. This difference in results would be due firstly to the agro-climatic and soil conditions of the countries in which the experiments were conducted and secondly to the extract and the type of animals used. Moreover, white blood cells defend the body against pathogens and eliminate damaged cells and other waste products [47]. The decrease of white blood cells to below normal values could expose poultry to several diseases such as viral, bacterial and respiratory [48]. Thus, broilers in the negative control group were exposed mainly to bacterial diseases, which would explain the high mortality rate recorded in this treatment. Our results are in accordance with [24,26] who worked on *M. indica* and *T. triangulare* respectively.

Short-chain fatty acids (SCFAs) are the end products of the fermentation of prebiotic compounds by gut microbes [49]. They have been widely studied because of their mechanisms of improving the gut barrier and suppressing gut inflammation [13,14]. Our results showed that the treatments that received the leaf powders produced more SCFAs due to a significantly (p < 0.05) different amount of formic acid than the other treatments. Several studies showed that formic acid inhibits pathogenic bacteria such as *Campylobacter jejuni*, *Clostridium perfringens*, *Streptococcus suis*, and *Streptococcus pneumoniae* [50]. In addition [51], showed that formic acid performs better against *Salmonella* spp. and *E. coli.* The effectiveness of formic acid against *Salmonella* spp. and *E. coli* lies in its small molecular size and long chain length [52]. Furthermore, studies by Ref. [53] showed that formic acid has antibacterial properties. Our results also showed that the amount of acetic acid was zero in the cecum of broilers that did not receive the antibiotic (control-), but this acid was quantified in the cecum of broilers from the other treatments (p < 0.05). Several studies showed that the presence of acetic acid in the cecum of poultry is correlated with *Bifidobacterium*, *Sutterella* and *Lactobacillus* [54,55]. These bacteria are beneficial in many animal species because they produce natural bacteriocins [54]. Our study also showed that the concentration of butyric acid was significantly different (p < 0.05) in favour of the positive control group; 2 % *T. Triangulare* and 2 % *M. Indica*. The presence of butyric acid in the cecum proves that beneficial bacteria such as *Faecalibacteria*, *Roseburia*, *Coprococcus*, *Anaerostripes*, *Odoribactus* [[55], [56], [57]] are present in the cecum of broilers. Several studies showed that acetate and butyrate are the main SCFAs that lower the cecal pH and inhibit the growth of pathogens by promoting the growth of beneficial bacteria [58,59]. Our results on SCFA concentration are different to those found by Refs. [54,60]. Each of these authors showed respectively the effects of agavins, fibrous feed and insulin on the production of SCFAs in broilers. Indeed, the amounts of acetic and butyric acids they found were higher than what we found in the present study. This difference would be related to the types of broilers and the biomolecules in each product used in our experiments on the one hand and the climatic conditions of the countries on the other hand.

Due to competition with food sources, animal feeds are becoming expensive. This has spurred research into alternative feedstuffs, especially non conventional ingredients as potential sources of animal feed supplements [24]. reported that T. triangulare is cost effective for the replacement of soya bean meal and ground nut cake in broiler diet without deleterious impacts on performance [61]. showed that the poultry served 120 ml of *Telfaria occidentalis* leaves extract per litre of water for 8 weeks had best performance in terms of cost benefit ratio and cost of feed per kilogramme live weight grain. Using *Moringa oleifera* leaves in the diets of poultry is a real opportunity for traditional stockholders to improve at lower cost, not only the productivity and nutritional status of their birds but also their income [62]. Effects of peppermint leaves can result in higher economical efficiency in broiler meat production [63].

## 7 Conclusion

From this study, it can conclude that the incorporation of *T. triangulare* and *M. indica* in the feed of broiler chickens improved growth performance, haematological parameters, formic acid concentration and reduced mortality rates. This improvement could be due to the nutritional composition of these plants and the bioactive compounds they contain, which have a number of pharmacological uses. These plants could therefore replace antibiotics in the production of slow-growing broilers.

## Data availability statement

Data will be made available on request.

## Additional information

No additional information is available for this paper.

## CRediT authorship contribution statement

**Dossou Bruno Sodjinou:** Writing – review & editing, Writing – original draft, Software, Methodology, Formal analysis, Conceptualization. **Faya Leno Pierre:** Methodology, Conceptualization. **Millimono Germaine:** Methodology, Conceptualization. **Akpavi Sêmihinva:** Writing – review & editing, Writing – original draft, Validation, Supervision, Methodology, Conceptualization. **Tona Kokou:** Writing – original draft, Validation, Methodology. **Frédéric Houndonougbo Makpondji:** Writing – review & editing, Writing – original draft, Validation, Supervision, Methodology.

## Declaration of competing interest

The authors declare the following financial interests/personal relationships which may be considered as potential competing interests:SODJINOU Dossou Bruno reports financial support was provided by 10.13039/100004421World Bank Group. If there are other authors, they declare that they have no known competing financial interests or personal relationships that could have appeared to influence the work reported in this paper.

## References

[1] Murugkar H.V., Rahman H., Kumar A., Bhattacharyya D. (2005). Isolation, phage typing and antibiogram of Salmonella from man and animals in Northern India. Indian J. Med. Res..

[2] Dhama K., Latheef S.K., Mani S., Samad H.A., Karthik K., Tiwari R., Khan R.U., Alagawany M., Farag M.R., Alam G.M., Laudadio V., Tufarelli V. (2015). Multiple beneficial applications and modes of action of herbs in poultry health and production-a review. Int. J. Pharmacol..

[3] Scanes C.G. (2007). The global importance of poultry. Poultry Sci..

[4] Apata D.F. (2009). Antibiotic resistance in poultry. Int. J. Poultry Sci..

[5] Mund M.D., Khan U.H., Tahir U., Bahar -E., Fayyaz A. (2017). Antimicrobial drug residues in poultry products and implications on public health: a review. Int. J. Food Prop..

[6] Venkitanarayanan K., Kollanoor-Johny A., Darre M., Donoghue A., Donoghue D. (2013). Use of plant-derived antimicrobials for improving the safety of poultry products. Poultry Sci..

[7] Aronu C.J., Okolo C.C., Akubueze U.H. (2019). Dietary supplementation with aqueous extract of Talinum triangulare at different phases of broiler production and effect on growth, serum biochemistry and carcass quality. Indian J. Anim. Res..

[8] Mengesha M. (2012). The issue of feed-food competition and chicken production for the demands of foods of animal origin. Asian J. Poultry Sci..

[9] Jeong J.S., Kim I.H. (2014). Effect of Bacillus subtilis C-3102 spores as a probiotic feed supplement on growth performance, noxious gas emission, and intestinal microflora in broilers. Poultry Sci..

[10] Ikele O.M., Ezeonu I.M., Umeh C.N. (2020). Prebiotic roles of ocimum gratissimum extract in the control of colibacillosis in broilers. J. Anim. Health Prod..

[11] Gadde U., Kim W.H., Oh S.T., Lillehoj H.S. (2017). Alternatives to antibiotics for maximizing growth performance and feed efficiency in poultry: a review. Anim. Health Res. Rev..

[12] Windisch W., Schedle K., Plitzner C., Kroismayr A. (2008). Use of phytogenic products as feed additives for swine and poultry. J. Anim. Sci..

[13] Rothhammer V., Mascanfroni I.D., Bunse L., Takenaka M.C., Kenison J.E., Mayo L. (2016). Type I interferons and microbial metabolites of tryptophan modulate astrocyte activity and central nervous system inflammation via the aryl hydrocarbon receptor. Nat Med.

[14] Gibson G.R., Hutkins R.W., Sanders M.E., Prescott S.L., Reimer R.A., Salminen S.J., Karen S., Stanton C., Swanson K.S., Cani P.D., Verbeke K., Reid G. (2017). The international scientific association for probiotics and prebiotics (ISAPP) consensus statement on the definition and scope of prebiotics. Gastroenterol. Hepatol..

[15] Alloui M.N., Szczurek W., Świątkiewicz S. (2013). The usefulness of prebiotics and probiotics in modern poultry nutrition: a review. Ann. Anim. Sci..

[16] Krysiak K., Konkol D., Korczynski M. (2021). Overview of the use of probiotics in poultry production. Animals.

[17] Kikusato M., Xue G., Pastor A., Niewold T.A., Toyomizu M. (2021). Effects of plant-derived isoquinoline alkaloids on growth performance and intestinal function of broiler chickens under heat stress. Poultry Sci..

[18] Abd El-Hack M.E., El-Saadony M.T., Elbestawy A.R., Nahed A., Saad A.M., Salem H.M., El-Tarabily K.A. (2021). Necrotic enteritis in broiler chickens: disease characteristics and prevention using organic antibiotic alternatives−a comprehensive review. Poultry Sci..

[19] Alagawany M., El-Saadony M.T., Elnesr S.S., Farahat M., Attia G., Madkour M., Reda F. (2021). Use of lemongrass essential oil as a feed additive in quail's nutrition: its effect on growth, carcass, blood biochemistry, antioxidant and immunological indices, digestive enzymes and intestinal microbiota. Poultry Sci..

[20] Wakte P., Bhusari S., Patil A., Kandangire P., Gite S., Shinde D. (2012). Activity guided isolation of antioxidant compound from leaves of mangifera indica. Int. J. Pharm. Pharmaceut. Sci..

[21] Ikewuchi C.C., Ikewuchi J.C., Ifeanacho M.O. (2016). Bioactive phytochemicals in an aqueous extract of the leaves of Talinum triangulare. Food Sci. Nutr..

[22] Ilodibia C.V., Igboabuchi N.A. (2017). Evaluation of phytochemical and nutritional potential of Talinum triangulare (Jacq) leaf, stem and root on human health. Int. Jour. of Biol. Resea..

[23] Somkuwar D.O., Kamble V.A. (2013). Phytochemical screening of ethanolic extracts of stem, leaves, flower and seed kernel of Mangifera indica L. Int. J. Pharm. Biol. Sci..

[24] Nworgu F.C., Alikwe P.C.N., Egbunike G.N., Ohimain E.I. (2015). Economic importance and growth rate of broiler chickens fed with water leaf (Talinum triangulare) meal supplements. AJAEES.

[25] Ayoola A.A., Ekunseitan D.A., Muhammad S.B., Oguntoye M.A. (2020). The effects of extraction methods of Mangifera indica and Azadirachta indica bark on in vitro antimicrobial efficacy and performance of broiler chickens. J. World Poult. Res..

[26] Aka-Tanimo H.A., Oshibanjo D.O., Adelowo V.O., Akwashiki M.A., Azi I.W., Oguche C.J., Sani H., Afiniki Y.S. (2020). Growth performance, carcass characteristics and heamatology indices of broiler chicken fed graded levels of (Mangifera indica) mango leaf meal. Asian Journal of Research in Animal and Veterinary Sciences.

[27] Washburn K.W., Peavey R., Renwick G.M. (1980). Relationship of strain variation and feed restriction to variation in blood pressure and response to heat stress. Poultry Sci..

[28] Zhao G.P., Chen J.L., Zheng M.Q., Wen J., Zhang Y. (2007). Correlated responses to selection for increased intramuscular fat in a Chinese quality chicken line. Poultry Sci..

[29] Chisanga B., Zulu-Mbata O. (2018). The changing food expenditure patterns and trends in Zambia: implications for agricultural policies. Food Secur..

[30] Sodjinou D.B., Agbodan K.M.L., Akpavi S., Houndonougbo M.F. (2022). Inventory of ethno-botanical knowledge and indigenous perception of plants used in poultry farms in the Maritime region of Togo. International Journal of Medicinal Plants and Natural Products.

[31] Stella S., Vitale S.R., Stagno F., Massimino M., Puma A., Tomarchio C., Pennisi M.S., Tirrò E., Romano C., Di Raimondo F. (2022). Impact of different cell counting methods in molecular monitoring of chronic myeloid Leukemia Patients. Diagnostics.

[32] Jimoh M.A., Okunlola G.O., Wahab A.A., Oseni O.M., Rufai A.B. (2020). Proximate and mineral analysis and antinutrient and antimicrobial properties of Talinum triangulare (Talinaceae) and Celosia argentea (Amaranthaceae). Phytol. Balc..

[33] Princewill-Ogbonna I.L., Ogbonna P.C., Ogujiofor I.B. (2019). Proximate composition, vitamin, mineral and biologically active compounds levels in leaves of mangifera indica (mango), Persea americana (Avocado pea), and Annona muricata (Sour sop). J. Appl. Sci. Environ. Manag..

[34] Akubugwo I.E., Obasi N.A., Chinyere G.C., Ugbogu A.E. (2007). Nutritional and chemical value of Amaranthus hybridus L. leaves from Afikpo, Nigeria. Afr. J. Biotechnol..

[35] Yamamoto M., Sakiyama K., Kitamura K., Yamamoto Y., Takagi T., Sekiya S., Watanabe G., Taniguchi S., Ogawa Y., Ishizuka S. (2022). Development and regeneration of muscle, Tendon, and myotendinous junctions in striated skeletal muscle. Int. J. Mol. Sci..

[36] Minyang Fu, Dandan Peng, Tianxia Lan, Yuquan Wei, Xiawei Wei, Multifunctional regulatory protein connective tissue growth factor (CTGF): A potential therapeutic target for diverse diseases, Acta Pharm. Sin. B, Volume 12, Issue 4, Pages 1740-1760, ISSN 2211-3835, 10.1016/j.apsb.2022.01.007. PMC9279711 35847511

[37] Ali B.A., Alfa A.A., Tijani K.B., Idris E.T., Unoyiza U.S., Junaidu Y. (2020). Nutritional health benefits and bioactive compounds of mangifera indica L (mango) leaves methanolic extracts. Asian Plant Research Journal.

[38] Sultana B., Hussain Z., Asif M., Munir A. (2012). Investigation on the antioxidant activity of leaves, peels, stems bark, and kernel of mango (mangifera indica L.). J. Food Sci..

[39] Liang D., Zhou Q., Gong W., Wang Y., Nie Z., He H., Li J., Wu J., WuCh, Zhang J. (2011). Studies on the antioxidant and hepatoprotective activities of polysaccharides from Talinum triangulare. J. Ethnopharmacol..

[40] Afonso V., Champy R., Mitrovic D., Collin P., Lomri A. (2007). Reactive oxygen species and superoxide dismutases: role in joint diseases. Joint Bone Spine.

[41] Bruck R., Aeed H., Avni Y., Shirin H., Matas Z., Shahmurov M. (2004). Melatonin inhibits nuclear factor kappa B activation and oxidative stress and protects against thioacetamide induced liver damage in rats. J. Hepatol..

[42] Etim N.A.N., Akpabio U., Okpongete R.O., Offiong E.E.A. (2014). Do Diets affect haematological parameters of poultry?. Br. J. Appl. Sci. Technol..

[43] Crawford J.H., Chacko B.K., Kevil C.G., Patel R.P. (2004). The red blood cell and vascular function in health and disease. Antioxidants Redox Signal..

[44] Christopher M.M., Shooshtari M.P., Levengood J.M. (2004). Assessment of erythrocyte morphologic abnormalities in mallards with experimentally induced zinc toxicosis. Am. J. Vet. Res..

[45] Jomova K., Makova M., Alomar S.Y., Alwasel S.H., Nepovimova E., Kuca K., Rhodes C.J., Valko M. (2022). Essential metals in health and disease. Chem. Biol. Interact..

[46] Airaodion A.I., Ogbuagu E.O., Ekenjoku J.A., Ogbuagu U., Airaodion E.O. (2019). Haematopoietic potential of ethanolic leaf extract of Talinum triangulare in wistar rats. Asian Journal of Research in Biochemistry.

[47] Ashton N. (2007). Physiology of red and white blood cells. Anaesth. Intensive Care Med..

[48] Yehia N., Salem H.M., Mahmmod Y., Said D., Samir M., Mawgod S.A., Sorour H.K., AbdelRahman M.A.A., Selim S., Saad A.M., El-Saadony M.T., El-Meihy R.M., Abd El-Hack M.E., El-Tarabily K.A., Zanaty A.M. (2023). Common viral and bacterial avian respiratory infections: an updated review. Poultry Sci..

[49] Yang G.Q., Yin Y., Liu H.Y., Liu G.H. (2016). Effects of dietary oligosaccharide supplementation on growth performance, concentrations of the major odor-causing compounds in excreta, and the cecal microflora of broilers. Poultry Sci..

[50] Kovanda L., Zhang W., Wei X., Luo J., Wu X., Atwill E.R. (2019). In vitro antimicrobial activities of organic acids and their derivatives on several species of Gram-negative and Grampositive bacteria. Molecules.

[51] Gómez-García M., Sol C., Nova P.J.G., Puyalto M., Mesas L., Puente H. (2019). Antimicrobial activity of a selection of organic acids, their salts and essential oils against swine enteropathogenic bacteria. Porc Health Manag.

[52] Nikaido H. (2003). Molecular basis of bacterial outer membrane permeability revisited. Microbiol. Mol. Biol. Rev..

[53] Ricke S.C., Dittoe D.K., Richardson K.E. (2020). Formic acid as an antimicrobial for poultry production: a review. Front. Vet. Sci..

[54] Song J., Li Q., Everaert N., Liu R., Zheng M., Zhao G., Wen J. (2020). Dietary inulin supplementation modulates short-chain fatty acid levels and cecum microbiota composition and function in chickens infected with Salmonella. Front. Microbiol..

[55] Sun B., Hou L., Yang Y. (2020). Effects of altered dietary fiber on the gut microbiota, short-chain fatty acids and cecum of chickens during different growth periods. Preprints.

[56] Susan E.P., Duncan S.H., Hold G.L. (2002). The microbiology of butyrate formation in the human colon. FEMS (Fed. Eur. Microbiol. Soc.) Microbiol. Lett..

[57] Duncan S.H., Hold G.L., Harmsen H.J., Stewart C.S., Flint H.J. (2002). Growth requirements and fermentation products of Fusobacterium prausnitzii, and a pro-posal to reclassify it as Faecalibacterium prausnitzii gen. nov., comb. nov. Int. J. Syst. Evol. Microbiol..

[58] Teng P.Y., Kim W.K. (2018). Review: roles of prebiotics in intestinal ecosystem of broilers. Front. Vet. Sci..

[59] Micciche A.C., Foley S.L., Pavlidis H.O., McIntyre D.R., Ricke S.C. (2018). A review of prebiotics against Salmonella in poultry: current and future potential for microbiome research applications. Front. Vet. Sci..

[60] Curbelo Y.G., Salabarría R.B., Sanchez B.R., Dorta N.A., González A.A., Villafranca M.H., Pérez M.G.L. (2020). Prebiotic effects of agavins in poultry. Livest. Res. Rural Dev..

[61] Nworgu F.C. (2007). Economic importance and growth rate of broiler chickens served fluted pumpkin (Telfaria occidentalis) leaves extract. Afr. J. Biotechnol..

[62] Ayssiwede S.B., Dieng A., Bello H., Chrysostome C.A.A.M., Hane M.B., Mankor A., Dahouda M., Houinato M.R., Hornick J.L., Missohou (2011). Effects of moringa oleifera (Lam.) leaves meal incorporation in diets on growth performances, carcass characteristics and economics results of growing indigenous Senegal chickens. Pakistan J. Nutr..

[63] Abdel-Wareth A.A.A., Saskia K., Karl-Heinz S. (2019). Peppermint and its respective active component in diets of broiler chickens: growth performance, viability, economics, meat physicochemical properties, and carcass characteristics. Poultry Sci..

